# Predicting movement speed of beetles from body size and temperature

**DOI:** 10.1186/s40462-023-00389-y

**Published:** 2023-05-16

**Authors:** Jördis F. Terlau, Ulrich Brose, Thomas Boy, Samraat Pawar, Malin Pinsky, Myriam R. Hirt

**Affiliations:** 1 EcoNetLab, German Centre for Integrative Biodiversity Research (iDiv) Halle-Jena-Leipzig, Puschstraße 4, 04103 Leipzig, Germany; 2 Institute of Biodiversity, Friedrich‐Schiller‐University Jena, Jena, Germany; 3 Department of Life Sciences, Imperial College London, Silwood Park, Ascot, UK; 4 Department of Ecology, Evolution and Natural Resources, Rutgers University, New Brunswick, NJ USA

**Keywords:** Exploratory speed, Climate warming, Image-based tracking, Movement ecology, Ectotherms, Thermal response, Allometry

## Abstract

Movement facilitates and alters species interactions, the resulting food web structures, species distribution patterns, community structures and survival of populations and communities. In the light of global change, it is crucial to gain a general understanding of how movement depends on traits and environmental conditions. Although insects and notably Coleoptera represent the largest and a functionally important taxonomic group, we still know little about their general movement capacities and how they respond to warming. Here, we measured the exploratory speed of 125 individuals of eight carabid beetle species across different temperatures and body masses using automated image-based tracking. The resulting data revealed a power-law scaling relationship of average movement speed with body mass. By additionally fitting a thermal performance curve to the data, we accounted for the unimodal temperature response of movement speed. Thereby, we yielded a general allometric and thermodynamic equation to predict exploratory speed from temperature and body mass. This equation predicting temperature-dependent movement speed can be incorporated into modeling approaches to predict trophic interactions or spatial movement patterns. Overall, these findings will help improve our understanding of how temperature effects on movement cascade from small to large spatial scales as well as from individual to population fitness and survival across communities.

**Supplementary Information:**

The online version contains supplementary material available at 10.1186/s40462-023-00389-y.

## Background

Movement is the essential link of species to their environment and each other, and is therefore vital to sustain individual as well as population survival and fitness [33, 56]. On smaller scales, it mediates accessing spatially distributed or mobile resources [56] and is thus one of the major processes driving trophic interactions [37, 39, 60, 69]. On larger scales, movement is the elementary process that shapes the spatial distribution of species [44] and also connects populations, communities, and entire ecosystems [53, 68]. Current knowledge about the movement patterns and processes of larger vertebrates is more comprehensive than ever before [23, 37, 46, 57]. Contrary and despite the immense importance of insects to our ecosystems highlighted by the multitude of their diversity, abundance and functional roles [28, 78], we still lack systematic information on their movement behavior and dynamics [45, 48].

This gap in our understanding of insect movement is partially caused by the difficulties of applying tracking technologies to small organisms. Laboratory measurements using camera tracking can help overcome these limitations. While they cannot be used to assess natural movement patterns that depend on the environment like habitat structure or microclimates [74, 76], they can help gain a deepened understanding about movement parameters and fundamental movement capacities. This information can then be used to inform mechanistic models, which can support predictions of potential movement patterns in natural environments [36]. Such movement parameters include maneuverability or movement speed. Movement speed, for instance, captures the movement intensity and its body-size dependence [37, 38, 43], which allows generalizations from a few measured species to the multitude of other species in the wild. During attacks or escapes, animals move at maximum speed. In contrast, they use a more constant and less demanding routine speed during dispersal (travel speed, minimizing the energy costs or habitat exploration (exploratory speed; maximizing the energy gain [18]. The relative exploratory speed of interacting species, for instance, is the major constraint on encounter and subsequent consumption rates, and thus drives interaction strengths [39, 60].

Because many physiological and behavioral processes of insects such as metabolism [12, 14, 15, 25, 32, 34] or growth rates [30, 67] are strongly driven by ambient temperature, all higher level processes that arise from them such as demography and movement are also strongly temperature-dependent [29, 35, 71]. Yet, studies on the consequences of climate warming on insect movement remain challenging and scarce compared to less diverse taxa [24]. Hitherto, studies on the thermal sensitivity of movement have with some exceptions [41] mostly focused on vertebrates like lizards or other single species [3, 13, 16, 17], and we still lack information on these sensitivities across wider taxonomic and body size ranges. A general thermal scaling relationship of movement speed across different species and body sizes will, in the long term, help to gain a mechanistic understanding of how terrestrial insects will respond to climate warming.

Here, we contribute to filling this gap by assessing the general allometric and thermal response of exploratory speed of ground beetles. Coleoptera are the largest taxonomic group of insects and occur in almost every ecosystem [28]. The group of Carabids holds an important role as predators, fulfilling, for instance, the ecosystem service of biological control [24]. We assessed the movement of 125 individuals of eight Carabid beetle species varying by an order of magnitude in body size using automated image-based tracking [6, 19]. We hypothesized that exploratory speed should follow a power-law relationship with body mass and show a unimodal response to temperature. The main objective of this study was to yield a general allometric and thermodynamic equation to predict exploratory speed from temperature and body mass.

## Methods and materials

### Study organisms and experimental design

We measured the thermal response of exploratory speed of 125 individuals of eight Central European Carabid beetle species (Carabidae) in the laboratory using automated image-based tracking [6, 19]. We collected the beetles in the surrounding area of Leipzig, Saxony, Germany (51.2910° N, 12.3220° E and 51.2799° N, 12.4119° E) during 2018–2020 using pitfall traps. Thereby, we obtained the following species for our experiment: *Carabus granulatus, Carabus nemoralis, Pterostichus cristatus, Pterostichus melanarius, Abax parallelus*, *Nebria brevicollis, Harpalus affinis,* and *Anchomenus dorsalis* with body masses ranging from 10 mg (*Anchomenus dorsalis)* to 303 mg (*Pterostichus cristatus*). As our main objective was quantifying a general allometric and thermal response of movement speed, we grouped the species into body mass classes to get a representative number of replicates across body masses (see Additional file 1: Tables S1–S3). However, this approach inhibited species-specific analysis of thermal responses. We kept all species separately in boxes (30 × 40 cm) filled with soil, leaves, and bark as habitat structure. The boxes were kept in a room with daylight to maintain a natural circadian rhythm at an ambient temperature of ~ 19 °C. We fed beetles ad libitum with beetle jelly from a commercial supplier and watered the boxes with a spray bottle. The individuals were kept for a maximum of one week before measurements.

**Fig. 1:**
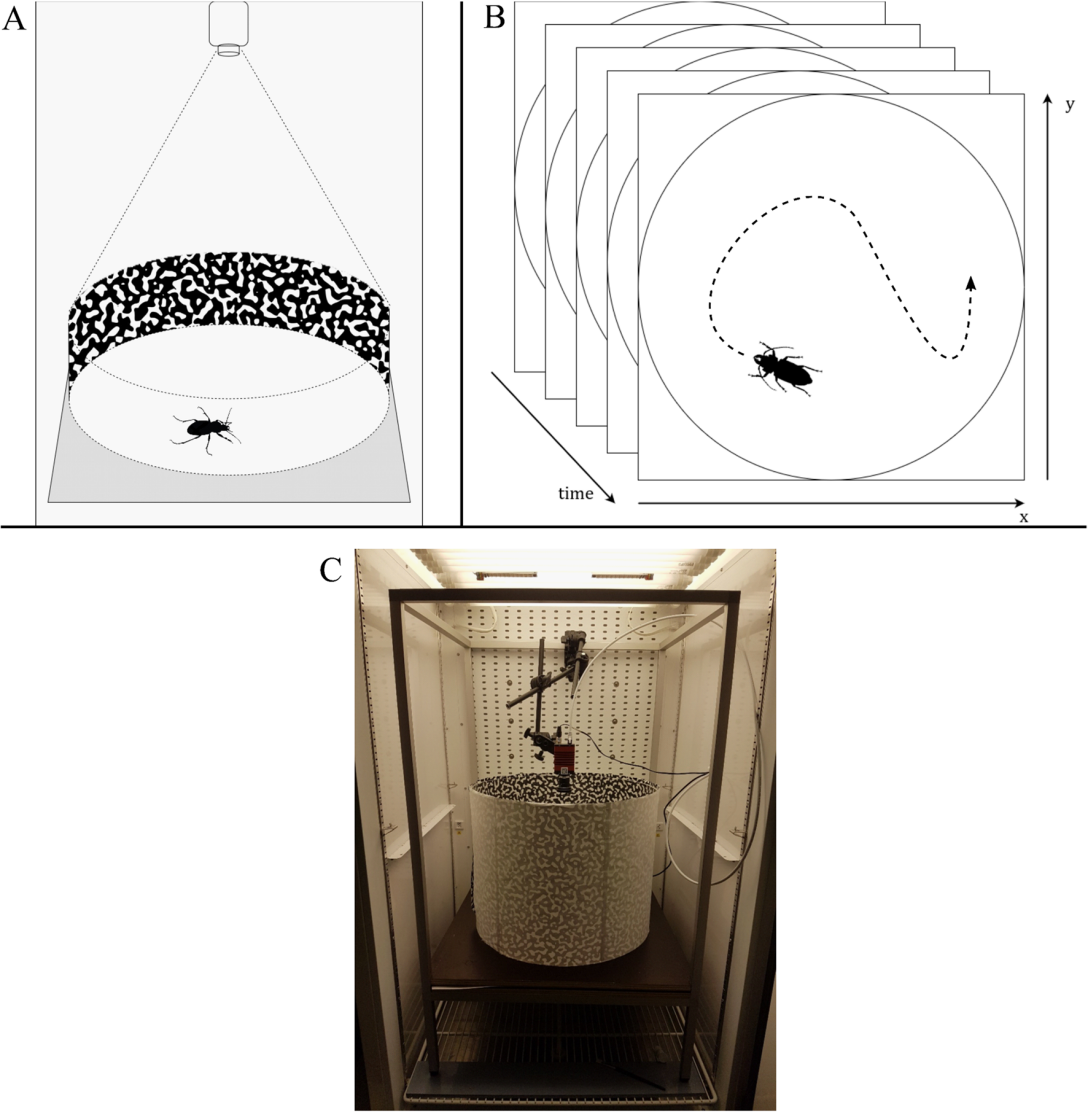
The experimental setup of the automated image-based tracking of beetles in an environmental reach-in chamber. **A** Sketch of the experimental setup. **B** Sketch of an automated image-based tracking sequence including x- and y-coordinates for each timestamp. **C** Actual experimental setup in an environmental reach-in chamber

For the filming records, we used two reach-in environmental chambers in which we placed circular acrylic-tubes of 490 mm diameter as arenas (Fig. 1). To create a non-uniform background and to avoid a directional bias of moving beetles, we covered the sides with a random black-white pattern. Additionally, we applied insect escape protection lacquer (Polytetrafluorethen) on the first 4 cm of the acrylic tube to prevent the beetles from climbing up the arena wall. We located a high-resolution camera (Prosilica GT 1920; Allied Vision; 1936 × 1454 pixel) orthogonally above the arena. The bottom of each arena was covered with white paper (80 g/m^2^), which was exchanged every new day of recording or when a different species was recorded. We tracked a maximum of three individuals per day and per environmental chamber. To track the beetles, we used an open-source software application (Vimba-Viewer) using the C +  + framework of the camera producer (Allied Vision) at a frame rate of 38 pictures per seconds. The internal real time clock of the camera provided high precision timestamps for every frame. We developed C +  + applications and scripts for extracting movement trajectories with real world coordinates and timestamps [10]. We analyzed the trajectory data, which consists of x–y-coordinates and time stamps using the R-package trajr [54]. Prior experiments with non-moving animals showed that artificial changes in position and direction may be recorded although the beetle was inactive [38]. To remove these spurious movement periods, we set thresholds and excluded movement data if speeds were lower than 0.6 mm/s (start) and 0.3 mm/s (stop). Before starting a film recording session, we weighed each individual and kept the beetles separately in small boxes with perforated lids and added beetle jelly to the boxes to make sure that all beetles were in the same condition and well fed before starting the measurements. Following an acclimation time of two hours in the environmental chamber at the respective temperature, we released one single beetle into the arena per session. After a time delay of ten minutes to account for the temporarily open doors of the climate chamber, a one-hour film recording was initiated. We assume that a two hour acclimation time is sufficient to provide reliable results in our experiment. If, however, longer acclimation times would be needed, we can expect a slight underestimation of movement speed in our results.

We used a temperature gradient of 14 levels from 8 to 32 °C. This temperature range was limited by the technical constraints of the environmental reach-in chamber and the high-resolution camera and does therefore not capture very low temperatures like they occur in nature (see Additional file 1: Table S5). However, the highest temperature level of 32 °C still meets realistic temperatures in the environment of species occurrences (Additional file 1: Table S4). During the recording, we kept a constant temperature and took three separate records for every temperature level using different individuals. In total, we recorded movement, weight, and temperature data for 125 individuals across eight species.

### Analyses and statistics

To analyze the thermal response of movement speed, we fitted thermal performance curves (TPC) to our data by applying the nls_multstart function from the rTPC package [59]. Although different species will show variations in e.g. thermal optima, our main goal here was to predict the average thermal response across our species. We compared five different models included in this package, which we assumed as most relatable to our movement data [1]: *Gaussian, Modified Gaussian, Quadratic, Pawar* (a modified Sharpe-Schoolfield equation; [50] and *Weibull*. We compared these models by using the Akaike information criterion (AIC) to find the most parsimonious model. Based on the best model fit, we chose the respective equation and incorporated an additional power-law scaling with body mass [38], which yielded a final equation for predicting the exploratory speed from body mass and temperature. We used the nls function in R to fit the respective equation to our data.

Since we did not have sufficient individuals from all species to measure every species equally often across all temperature levels, we aggregated them in size classes (Additional file 1: Table S1–S3). Therefore, we could not test for species-specific responses or thermal optima. To account for species-specific responses, we used a linear model to test how the residuals of the general scaling model (exploratory speed depending on body mass and temperature, see above) vary with species identities as well as their habitat preferences (see Additional file 1: Table S1).

All statistical analyses and calculations were performed using R 4.2.1 [61]. We used the following R-packages for the graphical presentation: ggplot2 [77], grafify [72], and sjPlot [52].

## Results

**Fig. 2:**
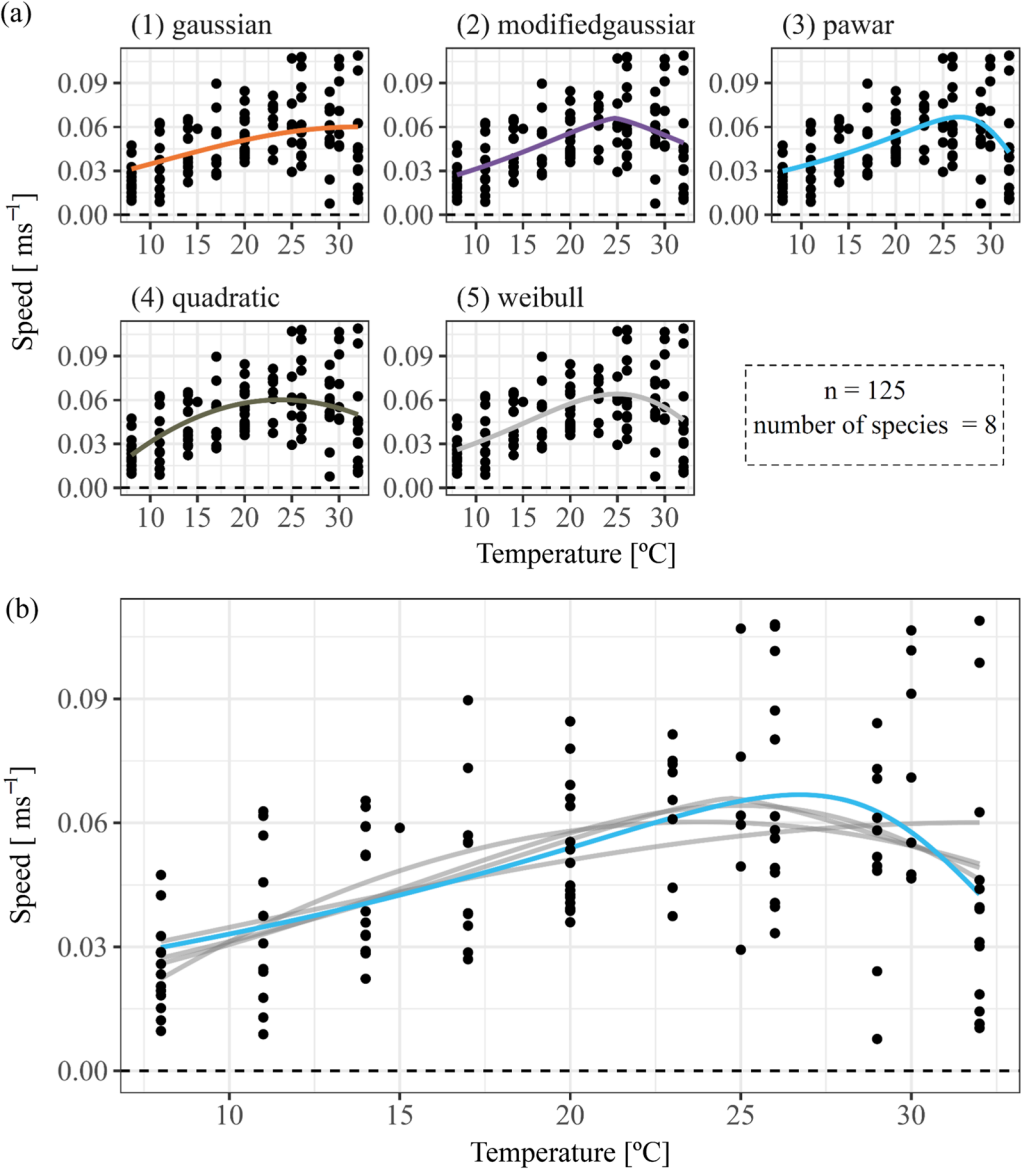
The unimodal scaling of exploratory speed [ms^−1^] with temperature [°C] of carabid beetles (n = 125, number of species = 8). **A** Five different thermal performance models included in the rTPC package [59] in comparison. **B** The final selected model based on AIC comparison (Table 2), a modified Sharpe-Schoolfield equation (pawar model, [50], blue curve). Gray curves show the other tested models in comparison

We measured movement speed of in total 125 individuals of ground beetles ranging between a body mass of 10 mg and 303 mg with an average body mass of 105 mg. The measured movement speed lay between 0.008 ms^−1^ and 0.11 ms^−1^. The data showed much variation (Fig. 2), which we aimed to explain by allometric and temperature effects. Subsequently, we carried out a sensitivity analysis on the residuals of this general scaling relationship to detect indications of species-specific responses (e.g., species-specific habitat and also thermal preferences).

The main goal of our study was to predict the general allometric and thermal response of exploratory speed across the species of our experiment. The thermal performance models we tested provided fairly similar fits to the data (Fig. 2B). AIC comparisons identified the *Pawar* model [50] and the *Weibull* model as the most parsimonious models (Table 1). We chose the *Pawar* model, a modified Sharpe-Schoolfield equation (frequently used to quantify the thermal response of ecological processes; [70], with the lowest AIC (delta AIC < 1.18) for all further analyses.

**Table 1:** AIC comparison of five thermal performance models included in the rTPC package [59] for movement speed [ms^−1^]

Model name	AIC	ΔAIC
Gaussian	− 591.13	11.75
Modified Gaussian	− 600.46	2.42
Quadratic	− 600.26	2.62
Weibull	− 601.70	1.18
Pawar	− 602.88	0

We modified the *Pawar* model (the modified Sharpe-Schoolfield equation; [50] by adding a body mass term, which yielded the following equation:

1 $$v = a_{0} M^{b} \cdot \frac{{exp^{{\frac{ - E }{k} \left( {\frac{1}{T + 273.15} - \frac{1}{{T_{ref} + 273.15}}} \right)}} }}{{1 + \left( {\frac{E}{{E_{h} - E}}} \right) \cdot exp^{{\frac{{E_{h} }}{k} \left( {\frac{1}{{T_{opt} + 273.15}} - \frac{1}{T + 273.15}} \right)}} }}$$

describing how movement speed *v* [m s^−1^] depends on body mass *M* [mg] and temperature *T* (°C). Here, the intercept *a*_*0*_ represents the movement speed at the reference temperature *T*_*ref*_ (here: 15 °C) and *b* is the allometric exponent. *E* is the activation energy (eV), which controls the rise of the curve up to the peak, *E*_*h*_ is the de-activation energy (eV), which sets the rate at which movement speed decreases after the peak, *k* is the Boltzmann constant (8.617 ⋅ 10^−5^ eV K^−1^), and *T*_*opt*_ is the optimum temperature at which movement speed is maximized (across species). Note that species-specific temperature optima likely vary, but could not be accurately predicted based on our data. Detailed information on the number of individuals per species, respective body-mass levels and the number of measurements per species and temperature treatment can be found in the Additional file 1: Tables S1–S3.

**Fig. 3:**
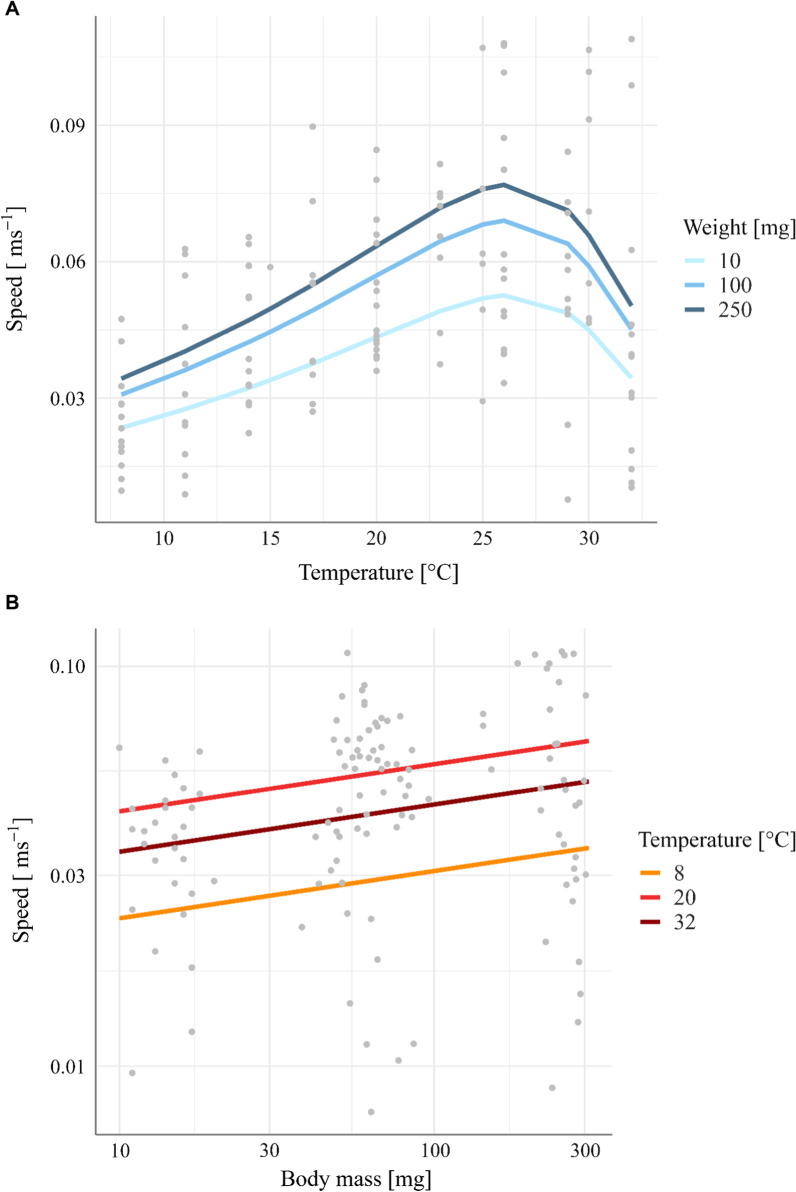
**A** The predicted scaling of movement speed [ms^−1^] with temperature [°C] for three different body masses [mg] (blue color scale) based on Eq. (1). **B** The predicted scaling of movement speed [ms^−1^] with body mass [mg] for three different temperature levels [°C] (orange-dark red color code)

To illustrate both temperature and body-size effects, we used our allometric and thermodynamic equation to predict movement speed [ms^−1^] for different body masses [mg] (across the temperature gradients) or at different temperature levels (across the body size gradient) temperature levels [°C]. Our results demonstrate a continuous increase in exploratory speed with body mass (Fig. 3B). Since a power law scaling with body mass with an exponent less than one (i.e. *b* = 0.12 CI = 0.02 – 0.15) indicates that this increase is steeper from small to medium species than from medium to large species, medium and large species are at a given temperature quite similar in their exploratory speed (Fig. 3A, medium and dark blue lines at a given temperature), whereas small species are much slower (Fig. 3A, light blue line at a given temperature).

The scaling of exploratory speed with temperature exhibits a more complex unimodal pattern (Fig. 3A). Speeds are increasing from low to intermediate temperatures (Fig. 3B, orange versus red lines) but decreasing from intermediate to high temperatures (Fig. 3B, red versus dark red lines). These differences are reflected in the model parameters with an activation energy *E* of 0.37 eV for the increasing part and a deactivation energy *E*_*h*_ of 3.11 eV for the decreasing part of the unimodal relationship (Table 2). Overall, this implies a steady increase in exploratory speed with warming up to the optimum temperature that is followed by a sharp decrease (Fig. 3A).

**Table 2:** Parameter values for Eq. (1), the modified Sharpe-Schoolfield equation after Kontopoulos et al. [50] with an additional body-mass term

Predictors	Variable	Estimates	Std. Error	CI	*p*
intercept	*a*	0.03	0.004	0.02 – 0.04	< 0.001
body mass exponent	*b*	0.12	0.04	0.02 – 0.15	0.002
activation energy	*E*	0.37	0.09	0.19 – 0.55	< 0.001
deactivation energy	*E*_*h*_	3.11	1.33	0.41 – 5.60	0.021
optimum temperature	*T*_*opt*_	26.33	1.20	24.22 – 28.72	< 0.001
Observations		125			

Subsequently, we carried out a sensitivity analysis using linear models to test how the residuals of the general scaling model (Fig. 3, Table 2) depend either on species identities or on their habitat preferences. Here, we tested whether the residuals for any group defined by either species identity (i.e. taxonomy) or habitat preference (i.e. species grouped by their habitat preferences) deviate significantly from zero representing the model prediction. These analyses did not show any significant effects of species identities (Additional file 1: Table S6, Figure S1) or habitat preferences (Additional file 1: Tables S7, Figure S2). Overall, these sensitivity analyses show that deviations of our empirical data points from our model predictions cannot be explained by species identities or habitat preferences.

## Discussion

Despite their abundance and functional importance, we still know little about the thermal sensitivity of movement of insects. Here, we experimentally measured the movement of differently-sized beetles across a temperature-gradient using image-based tracking [6, 19]. Thereby, we provide an allometric and thermodynamic model for predicting exploratory speed from body size and temperature.

Similar to Hirt et al. [38] we found a power-law scaling of exploratory speed with body mass with a slightly smaller allometric exponent (0.12 ± 0.04 compared to 0.19 ± 0.04; [38]. To account for the temperature-dependence of movement speed [3, 13, 17], we fitted a thermal performance curve to our data, which was best described by the modified Sharpe-Schoolfield equation [50]. While some of the variation in the measured speed data finds an explanation in body mass effects (Fig. 3A) or temperature effects (Fig. 3B) that are both accounted for by our fitted model (Eq. 1, Table 2), there is also unexplained variation that is potentially related to species-specific responses. Analyses of effects resulting from species and habitat preferences on residuals showed no significant effects (Additional file 1: Tables S6–S7, Figures S1–S2). This suggests that in our data set, species identities and habitat preferences do not contribute towards explaining variation in exploratory speed after accounting for the effects of body mass and ambient temperature. Nevertheless, we caution that larger datasets covering more species may find signatures of species-specific effects. In particular, our sensitivity test for species-specific effects was inspired by findings of shorter acclimation times for smaller animals also making larger animals more sensitive to higher temperatures [49, 64]. Additionally, thermal performance generally depends on age (life-history stage), body size and geographic location [58]. Since all individuals of our study were collected within the same area around Leipzig (Germany), we can assume that the species in our study should not differ much regarding adaptation to the geographic location in general, but rather regarding their species-specific habitat preferences (Additional file 1: Table S1) and hence respective microclimatic preferences [8]. As thermal responses generally vary among species and even populations [9, 55, 58], incorporating species-specific responses should be addressed in future research employing individuals or species from different geographic origins and climatic regimes in their habitats. Extending our approach across species from different biomes would be important for global predictions of the consequences of warming for animal movement.

Our general model relating animal exploratory speed to body mass and ambient temperature has broad implications for ecological processes*.* Movement speed is a crucial movement trait that strongly affects interactions, habitat connectivity, species distributions, and ultimately survival capacities of animals. The allometric and thermodynamic dependency of movement speed shown here has thus broad implications on small- and large-scale processes by implying that (1) larger animals have higher movement rates and (2) higher temperatures have variable effects on movement speed depending on the initial climatic conditions. While animals living in areas where they have not yet reached their optimal temperature will respond with higher average movement speeds to warming, animals from warmer climates that already live at or beyond their optimal temperature, will exhibit lower average movement speeds.

On smaller scales, higher movement speed as induced by higher body sizes or partially higher temperatures, should lead to higher encounter rates between predator and prey [60]. These higher encounter rates in turn yield higher attack rates and ultimately feeding rates [62]. Thus, together with prey preferences and prey density, movement speed is an important driver of interaction strengths and has direct consequences for energy fluxes (i.e., energy consumption across trophic groups) within food webs and therefore communities [5, 11]. With changing environments (e.g. due to climate warming), studies have found shifts in distribution patterns and habitat use [26, 51, 73, 75], which imply restructured food webs, including new as well as lost interaction links, and therefore altered interaction structure and strength of a whole food web [7]. The fact that both distribution shifts and consequently changes in species composition as well as the resulting local interactions depend on movement capacities, highlights the importance of understanding the trait-based response of movement to temperature to predict future communities re-shuffled by climate change.

On larger scales, higher movement speeds should on average result in higher travel distances of bigger species and thereby increase the connectivity of habitats and the linkage to other populations, species, or resources [36, 65]. This habitat connectivity could even increase under climate warming for species living in temperate regions but be detrimentally disrupted in warmer or colder climates depending on the relative temperature increase [63] and the thermal sensitivity of species [2, 23]. Since anthropogenic global change also causes disturbances such as habitat modification or fragmentation [66], our results suggests that under future conditions, larger animals living in temperate environments will be capable of longer travel distances to find new habitats and resources, whereas their movement capacity may become more limited in warm (e.g., tropic or Mediterranean) environments, which has strong consequences for their individual fitness and also survival of populations [22]. However, trophic interactions not only play a crucial role for the survival of individuals and populations, but also gene flow between populations, which is particularly achieved by dispersal [4]. Overall, the unimodal response of movement speed to warming will have opposing and cascading effects on individual fitness, species interactions, food webs, and species distributions.

The negative effects of warming on movement speed, however, can also be mitigated in nature, which cannot be captured under laboratory conditions like in our study. These coping mechanisms include either reducing movement or seeking shelter (shadow) and thereby lowering the overall energy loss [47, 74] or shifting activity periods (seasonal and diurnal). This, however, can potentially create activity mismatches between trophic levels, hence imposing cascading effects across food webs [71], which highlights the importance of considering the combined effects of temperature and habitat structure on movement speed and behavior in more complex experimental settings or field studies. Our thermal and allometric scaling relationships can serve as a baseline for these studies.

Since small invertebrates are hard to track and monitor, trait-based modeling approaches can be a powerful tool to make predictions on the general effects of warming on invertebrate movement. Integrating our equation in such models could enable predictions on trophic interactions or spatial patterns. For instance, biological rates like metabolism and growth also show a temperature- and body-mass dependence. These processes interactively drive energy gains via feeding and losses via metabolic expenditure and thus determine the energetic capacity of animals. Regarding ongoing and fast proceeding climate change, it raises the question how animals will energetically cope with increasing temperature and more often heat extremes [27]. If, for instance, energy loss increases faster than energy intake (i.e., feeding), this would create energetic discrepancies [40]. Thus, a synthesis approach integrating physiological rates and movement as a central process of species interactions may provide important insights in animal survival capacities under climate warming. Therefore, however, it is important to measure the thermal response of movement speed across a wider range of taxonomic groups, which would also allow testing for differences due to taxonomic traits, mode of locomotion, diet or ecological requirements. This would also include taxa from different climatic regions since we would expect varying thermal responses depending on the initial climatic condition [20, 21]. Similar to other studies (e.g., [31], we were unable to measure a temperature gradient covering the entire thermal performance gradient of all species due to technical limitations. If future studies could extend this temperature range further, it would improve our predictions, especially at the lower and upper critical temperature limits.

## Conclusions

Movement speed is an essential movement trait of animals shaping central ecological patterns and processes, making it important to understand how it will be altered by global change drivers such as climate warming. Although insects and Coleoptera in particular represent the largest taxonomic group, we still know little about the effects of climate change on this huge and ecologically important group. Our experimental approach provides a mathematical equation for predicting movement speed of Central European ground beetles (Carabidae) from temperature and body mass. This equation can be used to inform modeling approaches and will thereby help to better understand and predict the consequences of warming on species interactions, food web structures, species distribution patterns, and therefore ultimately survival of populations and communities.

## Supplementary Information

**Additional file 1**. Supplementary tables and figures.

## Data Availability

Should the manuscript be accepted, the data will be archived in an appropriate public repository and the DOI will be included at the end of the article.
